# Cognitive Behavioral Therapy for Eating Disorders

**DOI:** 10.1016/j.psc.2010.04.004

**Published:** 2010-09

**Authors:** Rebecca Murphy, Suzanne Straebler, Zafra Cooper, Christopher G. Fairburn

**Affiliations:** Department of Psychiatry, Warneford Hospital, Warneford Lane, Oxford University, Oxford OX3 7JX, UK

**Keywords:** Cognitive behavioral therapy, Eating disorders, Anorexia nervosa, Bulimia nervosa

## Abstract

Cognitive behavioral therapy (CBT) is the leading evidence-based treatment for bulimia nervosa. A new “enhanced” version of the treatment appears to be more potent and has the added advantage of being suitable for all eating disorders, including anorexia nervosa and eating disorder not otherwise specified. This article reviews the evidence supporting CBT in the treatment of eating disorders and provides an account of the “transdiagnostic” theory that underpins the enhanced form of the treatment. It ends with an outline of the treatment's main strategies and procedures.

The eating disorders provide one of the strongest indications for cognitive behavioral therapy (CBT). Two considerations support this claim. First, the core psychopathology of eating disorders, the overevaluation of shape and weight, is cognitive in nature. Second, it is widely accepted that CBT is the treatment of choice for bulimia nervosa^1^ and there is evidence that it is as effective with cases of “eating disorder not otherwise specified” (eating disorder NOS),^2^ the most common eating disorder diagnosis. This article starts with a description of the clinical features of eating disorders and then reviews the evidence supporting cognitive behavioral treatment. Next, the cognitive behavioral account of eating disorders is presented and, last, the new “transdiagnostic” form of CBT is described.

## Eating disorders and their clinical features

### Classification and Diagnosis

Eating disorders are characterized by a severe and persistent disturbance in eating behavior that causes psychosocial and, sometimes, physical impairment. The DSM-IV classification scheme for eating disorders recognizes 2 specific diagnoses, anorexia nervosa (AN) and bulimia nervosa (BN), and a residual category termed eating disorder NOS.^3^

The diagnosis of anorexia nervosa is made in the presence of the following features:

1. The overevaluation of shape and weight; that is, judging self-worth largely, or even exclusively, in terms of shape and weight. This has been described in various ways and is often expressed as strong desire to be thin combined with an intense fear of weight gain and fatness.
2. The active maintenance of an unduly low body weight. This is commonly defined as maintaining a body weight less than 85% of that expected or a body mass index (BMI; weight kg/height m^2^ or weight lb/[height in]^2^ × 703) of 17.5 or less.
3. Amenorrhea, in postpubertal females not taking an oral contraceptive.

The unduly low weight is pursued in a variety of ways with strict dieting and excessive exercise being particularly prominent. A subgroup also engages in episodes of binge eating and/or “purging” through self-induced vomiting or laxative misuse.

For a diagnosis of bulimia nervosa 3 features need to be present:

1. Overevaluation of shape and weight, as in anorexia nervosa.
2. Recurrent binge eating. A “binge” is an episode of eating during which an objectively large amount of food is eaten for the circumstances and there is an accompanying sense of loss of control.
3. Extreme weight-control behavior, such as recurrent self-induced vomiting, regular laxative misuse, or marked dietary restriction.

In addition, the diagnostic criteria for anorexia nervosa should not be met. This “trumping rule” ensures that patients do not receive both diagnoses at one time.

There are no positive criteria for the diagnosis of eating disorder NOS. Instead, this diagnosis is reserved for eating disorders of clinical severity that do not meet the diagnostic criteria of AN or BN. Eating disorder NOS is the most common eating disorder encountered in clinical settings constituting about half of adult outpatient eating-disordered samples, with patients with bulimia nervosa constituting about a third, and the rest being cases of anorexia nervosa.^4^ In inpatient settings the great majority of cases are either underweight forms of eating disorder NOS or anorexia nervosa.^5^

In addition, DSM-IV recognizes “binge eating disorder” (BED) as a provisional diagnosis in need of further study. The criteria for BED are recurrent episodes of binge eating in the absence of extreme weight-control behavior. It is proposed that BED be recognized as a specific eating disorder in DSM-V.^6^

### Clinical Features

Anorexia nervosa, bulimia nervosa, and most cases of eating disorder NOS share a core psychopathology: the overevaluation of the importance of shape and weight and their control. Whereas most people judge themselves on the basis of their perceived performance in a variety of domains of life (such as the quality of their relationships, their work performance, their sporting prowess), for people with eating disorders self-worth is dependent largely, or even exclusively, on their shape and weight and their ability to control them. This psychopathology is peculiar to the eating disorders (and to body dysmorphic disorder).

In anorexia nervosa, patients become underweight largely as a result of persistent and severe restriction of both the amount and the type of food that they eat. In addition to strict dietary rules, some patients engage in a driven form of exercising, which further contributes to their low body weight. Patients with anorexia nervosa typically value the sense of control that they derive from undereating. Some practice self-induced vomiting, laxative and/or diuretic misuse, especially (but not exclusively) those who experience episodes of loss of control over eating. The amount of food eaten during these “binges” is often not objectively large; hence, they are described as “subjective binges.” Many other psychopathological features tend to be present, some as a result of the semistarvation. These include depressed and labile mood, anxiety features, irritability, impaired concentration, loss of libido, heightened obsessionality and sometimes frank obsessional features, and social withdrawal. There are also a multitude of physical features, most of which are secondary to being underweight. These include poor sleep, sensitivity to the cold, heightened fullness, and decreased energy.

Patients with bulimia nervosa resemble those with anorexia nervosa both in terms of their eating habits and methods of weight control. The main feature distinguishing these 2 groups is that in patients with bulimia nervosa attempts to restrict food intake are regularly disrupted by episodes of (objective) binge eating. These episodes are often followed by compensatory self-induced vomiting or laxative misuse, although there is also a subgroup of patients who do not purge (nonpurging bulimia nervosa). As a result of the combination of undereating and overeating the weight of most patients with bulimia nervosa tends to be unremarkable and is within the healthy range, BMI  =  20–25. Features of depression and anxiety are prominent in these patients. Certain of these patients engage in self-harm and/or substance and alcohol misuse and may attract the diagnosis of borderline personality disorder. Most have few physical complaints, although electrolyte disturbance may occur in those who vomit or take laxatives or diuretics frequently.

The clinical features of patients with eating disorder NOS closely resemble those seen in anorexia nervosa and bulimia nervosa and are of comparable duration and severity.^7^ Within this diagnostic grouping 3 subgroups may be distinguished, although there are no sharp boundaries among them. The first group consists of cases that closely resemble anorexia nervosa or bulimia nervosa but just fail to meet the threshold set by the diagnostic criteria (eg, binge eating may not be frequent enough to meet criteria for BN or weight may be just above the threshold in AN); the second and largest subgroup comprises cases in which the features of AN and BN occur in different combinations from that seen in the prototypic disorders—these states may be best viewed as “mixed” in character—and the third subgroup comprises those with binge-eating disorder. Most patients with binge-eating disorder are overweight (BMI = 25–30) or meet criteria for obesity (BMI ≥ 30).

## The empirical status of cognitive behavioral therapy for eating disorders

Consistent with the current way of classifying eating disorders, the research on their treatment has focused on the particular disorders in isolation. Wilson and colleagues^8^ have provided a narrative review of the studies of the treatment of the 2 specific eating disorders as well as eating disorder NOS, and an authoritative meta-analysis has been conducted by the UK National Institute for Health and Clinical Excellence (NICE).^1^ This systematic review is particularly rigorous and, as with all NICE reviews, it forms the basis for evidence-based guidelines for clinical management.

The conclusion from the NICE review, and 2 other recent systematic reviews,^9,10^ is that cognitive behavioral therapy (CBT-BN) is the clear leading treatment for bulimia nervosa in adults. However, this is not to imply that CBT-BN is a panacea, as the original version of the treatment resulted in only fewer than half of the patients who completed treatment making a full and lasting recovery.^8^ The new “enhanced” version of the treatment (CBT-E) appears to be more effective.^2^

Interpersonal psychotherapy (IPT) is a potential evidence-based alternative to CBT-BN in patients with bulimia nervosa and it involves a similar amount of therapeutic contact, but there have been fewer studies of it.^11,12^ IPT takes 8 to 12 months longer than CBT-BN to achieve a comparable effect. Antidepressant medication (eg, fluoxetine at a dose of 60 mg daily) has also been found to have a beneficial effect on binge eating in bulimia nervosa but not as great as that obtained with CBT-BN and the long-term effects remain largely untested.^13^ Combining CBT-BN with antidepressant medication does not appear to offer any clear advantage over CBT-BN alone.^13^ The treatment of adolescents with bulimia nervosa has received relatively little research attention to date.

There has been much less research on the treatment of anorexia nervosa. Most of the studies suffer from small sample sizes and some from high rates of attrition. As a result, there is little evidence to support any psychological treatment, at least in adults. In adolescents the research has focused mainly on family therapy, with the result that the status of CBT in younger patients is unclear.

Preliminary findings have been reported from a 3-site study of the use of the enhanced form of CBT (CBT-E) to treat outpatients with anorexia nervosa.^14^ This is the largest study of the treatment of anorexia nervosa to date. In brief, it appears that the treatment can be used to treat about 60% of outpatients with the disorder (BMI 15.0 to 17.5) and that in these patients about 60% have a good outcome. Interestingly and importantly the relapse rate appears low.

There is a growing body of research on the treatment of binge-eating disorder. This research has been the subject of a recent narrative review^15^ and several systematic reviews.^1,16,17^ The strongest support is for a form of CBT similar to that used to treat BN (CBT-BED). This treatment has been found to have a sustained and marked effect on binge eating, but it has little effect on body weight, which is typically raised in these patients. Arguably the leading first-line treatment is a form of guided cognitive behavioral self-help as it is relatively simple to administer and reasonably effective.^18^

Until recently, there had been almost no research on the treatment of forms of eating disorder NOS other than binge-eating disorder despite their severity and prevalence.^7^ However, recently the first randomized controlled trial of the enhanced form of CBT found that CBT-E was as effective for patients with eating disorder NOS (who were not significantly underweight; BMI >17.5) as it was for patients with bulimia nervosa with two-thirds of those who completed treatment having a good outcome.^2^

In summary, CBT is the treatment of choice for bulimia nervosa and for binge-eating disorder with the best results being obtained with the new “enhanced” form of the treatment. Recent research provides support for the use of this treatment with patients with eating disorder NOS and those with anorexia nervosa.

The remainder of this article provides a description of this transdiagnostic form of CBT.

## The cognitive behavioral account of eating disorders

Although the DSM-IV classification of eating disorders encourages the view that they are distinct conditions, each requiring their own form of treatment, there are reasons to question this view. Indeed, it has recently been pointed out that what is most striking about the eating disorders is not what distinguishes them but how much they have in common.^19^ As noted earlier, they share many clinical features, including the characteristic core psychopathology of eating disorders: the overevaluation of the importance of shape and weight. In addition, longitudinal studies indicate that most patients migrate among diagnoses over time.^20^ This temporal movement among diagnostic categories, together with the shared psychopathology, has led to the proposal that there may be limited utility in distinguishing among the disorders^19^ and furthermore that common “transdiagnostic” mechanisms may be involved in their maintenance.

The transdiagnostic cognitive behavioral account of the eating disorders^19^ extends the original theory of bulimia nervosa^21^ to all eating disorders. According to this theory, the overevaluation of shape and weight and their control is central to the maintenance of all eating disorders. Most of the other clinical features can be understood as resulting directly from this psychopathology. It results in dietary restraint and restriction; preoccupation with thoughts about food and eating, weight and shape; the repeated checking of body shape and weight or its avoidance; and the engaging in extreme methods of weight control. The one feature that is not a direct expression of the core psychopathology is binge eating. This occurs in all cases of bulimia nervosa, many cases of eating disorder NOS, and some cases of anorexia nervosa. The cognitive behavioral account proposes that such episodes are largely the result of attempts to adhere to multiple extreme, and highly specific, dietary rules. The repeated breaking of these rules is almost inevitable and patients tend to react negatively to such dietary slips, generally viewing them as evidence of their poor self-control. They typically respond by temporarily abandoning their efforts to restrict their eating with binge eating being the result. This in turn maintains the core psychopathology by intensifying patients' concerns about their ability to control their eating, shape, and weight. It also encourages more dietary restraint, thereby increasing the risk of further binge eating.

Three further processes may also maintain binge eating. First, difficulties in the patient's life and associated mood changes make it difficult to maintain dietary restraint. Second, as binge eating temporarily alleviates negative mood states and distracts patients from their difficulties, it can become a way of coping with such problems. Third, in patients who engage in compensatory purging, the mistaken belief in the effectiveness of vomiting and laxative misuse as a means of weight control results in a major deterrent against binge eating being removed.

In patients who are underweight, the physiological and psychological consequences may also contribute to the maintenance of the eating disorder. For example, delayed gastric emptying leads to feelings of fullness even after patients have eaten only modest amounts of food. In addition, the social withdrawal and loss of previous interests prevent patients from being exposed to experiences that might diminish the importance they place on shape and weight.

**Fig. 1:**
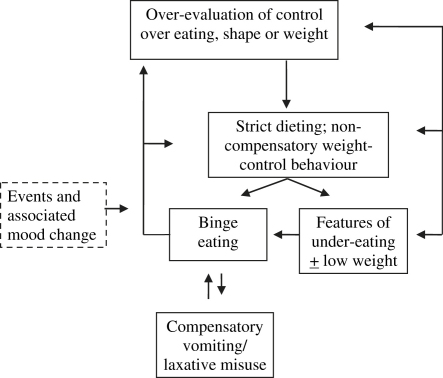
The composite “transdiagnostic” cognitive behavioral formulation.

The composite “transdiagnostic” formulation is shown in Fig. 1. This illustrates the core processes that are hypothesized to maintain the full range of eating disorders. When applied to individual patients, its precise form will depend on the psychopathology present. In some patients, most of the processes are in operation (for example, in cases of anorexia nervosa binge-purge subtype) but in others only a few are active (for example, in binge-eating disorder). Thus, for each patient the formulation is driven by their individual psychopathology rather than their DSM diagnosis. As such, the formulation provides a guide to those processes that need to be addressed in treatment.

## Enhanced cognitive behavioral therapy

“Enhanced” cognitive behavioral therapy (CBT-E) is based on the transdiagnostic theory outlined earlier and was derived from CBT-BN. It is designed to treat eating disorder psychopathology rather than an eating disorder diagnosis, with its exact form in any particular case depending on an individualized formulation of the processes maintaining the disorder. CBT-E is designed to be delivered on an individual basis to adult patients with any eating disorder of clinical severity who are appropriate to treat on an outpatient basis. It is described as “enhanced” because it uses a variety of new strategies and procedures to improve outcome and because it includes modules to address certain obstacles to change that are “external” to the core eating disorder, namely clinical perfectionism, low self-esteem, and interpersonal difficulties.

There are 2 forms of CBT-E. The first is the “focused” form (CBT-Ef) that exclusively addresses eating disorder psychopathology. Current evidence suggests that this form should be viewed as the “default” version, as it is optimal for most patients with eating disorders.^2^ The second, a broad form of the treatment (CBT-Eb), addresses external obstacles to change, in addition to the core eating disorder psychopathology. Preliminary evidence suggests that this more complex form of CBT-E should be reserved for patients in whom clinical perfectionism, core low self-esteem, or interpersonal difficulties are pronounced and maintaining the eating disorder.^2^

There are also 2 intensities of CBT-E. With patients who are not significantly underweight (BMI above 17.5), it consists of 20 sessions over 20 weeks. This version is suitable for the great majority of adult outpatients. For patients who have a BMI below 17.5, a commonly used threshold for anorexia nervosa, treatment involves 40 sessions over 40 weeks. The additional sessions and treatment duration are designed to allow sufficient time for 3 additional clinical features to be addressed, namely, limited motivation to change, undereating, and being underweight.

In addition CBT-E has been adapted for younger patients^22^ and for inpatient and day patient settings treatment.^23,24^ Limitations on space preclude a description of these other forms of CBT-E. Further details of these adaptations of CBT-E, together with a comprehensive account of the treatment and its implementation, can be found in the main treatment guide.^25^

## An overview of the core aspects of treatment

CBT-E is a form of cognitive behavioral therapy and in common with other empirically supported forms of CBT it focuses primarily on the maintaining processes, in this case those maintaining the eating disorder psychopathology. It uses specified strategies and a flexible series of sequenced therapeutic procedures to achieve both cognitive and behavioral changes. The style of treatment is similar to other forms of CBT, that of collaborative empiricism. Although CBT-E uses a variety of generic cognitive and behavioral interventions (such as addressing cognitive biases), unlike some forms of CBT, it favors the use of strategic changes in behavior to modify thinking rather than direct cognitive restructuring. The eating disorder psychopathology may be likened to a house of cards with the strategy being to identify and remove the key cards that are supporting the eating disorder, thereby bringing down the entire house. Following, we summarize the core features of the focused and broad versions of CBT-E, including adaptations that need to be made for patients who are underweight. The treatment has 4 defined stages.

## Preparation for treatment and change

An evaluation interview assessing the nature and extent of the patient's psychiatric problems is conducted before starting treatment.^26^ This interview usually takes place over 2 or more appointments. The assessment process is collaborative and designed to put the patient at ease and begin to engage the patient in treatment and in change. Information from the assessment informs how best to proceed and, in particular, whether CBT-E is appropriate. If CBT-E is deemed to be appropriate, the main aspects of the therapy are described and patients are encouraged to make the most of the opportunity to overcome their eating disorder.

It is important that from the outset of CBT-E the patient is in a position to make optimum use of treatment. For this reason any potential barriers to benefiting from CBT-E should be explored. Important contraindications to beginning treatment immediately are physical features of concern, the presence of severe clinical depression, significant substance abuse, major distracting life events or crises, and competing commitments. Such factors should be addressed first before embarking on treatment.

## Stage one

It is crucial that treatment starts well. This is consistent with evidence that the magnitude of change achieved early in treatment is a good predictor of treatment outcome.^27,28^ This initial intensive stage, designed to achieve initial therapeutic momentum, involves approximately 8 sessions held twice weekly over 4 weeks. The aims of this first stage are to engage the patient in treatment and change, to derive a personalized formulation (case conceptualization) with the patient, to provide education about treatment and the disorder, and to introduce and implement 2 important procedures: collaborative “weekly weighing” and “regular eating.” The changes made in this first stage of treatment form the foundation on which other changes are built.

### Engaging the Patient in Treatment and Change

Many patients with eating disorders are ambivalent about treatment and change. Getting patients “on board” with treatment is a necessary first step. Engagement can be enhanced by conducting the assessment of the eating disorder in a way that helps the patient to become involved in, and hopeful about, the possibility of change and encourages the patient to take “ownership” of treatment.

### Jointly Creating the Formulation

This is usually done in the first treatment session and is a personalized visual representation of the processes that appear to be maintaining the eating problem. The therapist draws out the relevant sections of Fig. 1 in collaboration with the patient, incorporating the patient's own experiences and words. It is usually best to start with something the patient wishes to change (eg, binge eating). The formulation helps patients to realize both that their behavior is comprehensible and that it is maintained by a series of interacting self-perpetuating mechanisms that are open to change. It is explained that “the diagram” provides a guide to what needs to be targeted in treatment if patients are to achieve a full and lasting recovery. At this early stage in treatment the therapist should explain that it is provisional and may need to be modified as treatment progresses and understanding of the patient's eating problem increases.

### Establishing Real-time Self-monitoring

**Fig. 2:**
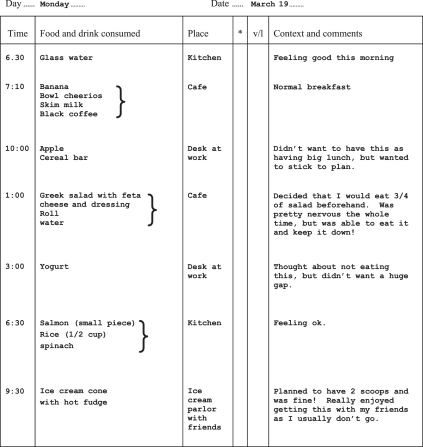
An example monitoring record.

This is the ongoing “in-the-moment” recording of eating and other relevant behavior, thoughts, feelings, and events (Fig. 2 is an example of a monitoring record). Self-monitoring is introduced in the initial session and continues to occupy an essential and central role throughout most of treatment. Therapists should clearly explain the reasons for self-monitoring. First, that it enables further understanding of the eating problem and it identifies progress. Second, and more importantly, it helps patients to be more aware of what is happening *in the moment* so that they can begin to make changes to behavior that may have seemed automatic or beyond their control. Fundamental to establishing accurate recording is jointly reviewing the patient's records each session and discussing the process of recording and any difficulties with this. The records also help inform the agenda for the session: it is best to save any problems identified in the records for the main part of the session.

### Establishing Collaborative “Weekly Weighing”

The patient and therapist check the patient's weight once a week and plot it on an individualized weight graph. Patients are strongly encouraged not to weigh themselves at other times. Weekly in-session weighing has several purposes. First, it provides an opportunity for the therapist to educate patients about body weight and help patients to interpret the numbers on the scale, which otherwise they are prone to misinterpret. Second, it provides patients with accurate data about their weight at a time when their eating habits are changing. Third, and most importantly, it addresses the maintaining processes of excessive body weight checking or its avoidance.

### Providing Education

From session 1 onward, an important element of treatment is education about weight and eating, as many patients have misconceptions that maintain their eating disorder. Some of the main topics to cover are as follows:

• The characteristic features of eating disorders including their associated physical and psychosocial effects
• Body weight and its regulation: the body mass index and its interpretation; natural weight fluctuations; and the effects of treatment on weight
• Ineffectiveness of vomiting, laxatives, and diuretics as a means of weight control
• Adverse effects of dieting: the types of dieting that promote binge eating; dietary rules versus dietary guidelines.

To provide reliable information on these topics, patients are asked to read relevant sections from one of the authoritative books on eating disorders^29,30^ and their reading is discussed in subsequent treatment sessions.

### Establishing “Regular Eating”

Establishing a pattern of regular eating is fundamental to successful treatment whatever the form of the eating disorder. It addresses an important type of dieting (“delayed eating”); it displaces most episodes of binge eating; it structures people's days and, for underweight patients, it introduces meals and snacks that can be subsequently increased in size. Early in treatment (usually by the third session) patients are asked to eat 3 planned meals each day plus 2 or 3 planned snacks so that there is rarely more than a 4-hour interval between them. Patients are also asked to confine their eating to these meals and snacks. They should choose what they eat with the only condition being that the meals and snacks are not followed by any compensatory behavior (eg, self-induced vomiting or laxative misuse). The new eating pattern should take precedence over other activities but should not be so inflexible as to preclude the possibility of adjusting timings to suit the patients' commitments each day.

Patients should be helped to adhere to their regular eating plan and to resist eating between the planned meals and snacks. Two rather different strategies may be used to achieve the latter goals. The first involves helping patients to identify activities that are incompatible with eating and likely to distract them from the urge to binge eat (eg, taking a brisk walk) and strategies that make binge eating less likely (eg, leaving the kitchen). The second is to help patients to recognize that the urge to binge eat is a temporary phenomenon that can be “surfed.” Some “residual binges” are likely to persist, however, and these are addressed later.

### Involving Significant Others

The treatment is primarily an individual treatment for adults. Despite this, “significant others” are seen if this is likely to facilitate treatment and the patient is willing for this to happen. There are 2 reasons for seeing others: if they could help the patient in making changes or if others are making it difficult for the patient to change, for example, by commenting adversely on eating or appearance.

## Stage two

Stage two is a brief, but essential, transitional stage that generally comprises 2 appointments, a week apart. While continuing with the procedures introduced in Stage one, the therapist and patient take stock and conduct a joint review of progress, the goal being to identify problems still to be addressed and any emerging barriers to change, to revise the formulation if necessary, and to design Stage three. The review serves several purposes. If patients are making good progress they should be praised for their efforts and helpful changes reinforced. If patients are not doing well, the explanation needs to be understood and addressed. If clinical perfectionism, core low self-esteem or relationship difficulties appear to be responsible, this would be an indication for implementing the broad version of the treatment.

## Stage three

This is the main body of treatment. Its aim is to address the key processes that are maintaining the patient's eating disorder. The mechanisms addressed, and the order in which these are tackled, depend upon their role and relative importance in maintaining the patient's psychopathology. There are generally 8 weekly appointments.

### Addressing the Overevaluation of Shape and Weight

#### Identifying the overevaluation and its consequences

**Fig. 3:**
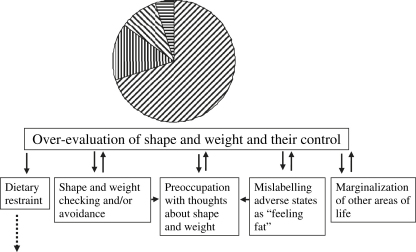
The overevaluation of shape and weight and their control: an extended formulation.

The first step involves explaining the concept of self-evaluation and helping patients identify how they evaluate themselves. The relative importance of the various domains that are relevant may be represented as a pie chart (Fig. 3 is an example of a pie chart with extended formulation), which for most patients is dominated by a large slice representing shape and weight and controlling eating.

The patient and therapist then identify the problems inherent in this scheme for self-evaluation. Briefly there are 3 related problems: first, self-evaluation is overly dependent on performance in one area of life with the result that domains other than shape and weight are marginalized; second, the area of controlling shape and weight is one in which success is elusive, thus undermining self-esteem; and third, the overevaluation is responsible for the behavior that characterizes the eating disorder (dieting, binge eating, and so forth).^31^

The final step in the consideration of self-evaluation is the creation of an “extended formulation” depicting the main expressions of the overevaluation of shape and weight: dieting, body checking and body avoidance, feeling fat, and marginalization of other areas of life. The therapist uses this extended formulation to explain how these behaviors and experiences serve to maintain and magnify the patient's concerns about shape and weight and thus they need to be addressed in treatment.

#### Enhancing the importance of other domains for self-evaluation

An indirect, yet powerful, means of diminishing the overevaluation of shape and weight is helping patients increase the number and significance of other domains for self-evaluation. Engaging in other aspects of their life that may have been pushed aside by the eating disorder results in these other areas becoming more important in the patient's self-evaluation. Briefly, this involves identifying activities or areas of life that the patient would like to engage in and helping them do so.

A second, direct, strategy is to target the behavioral expressions of the overevaluation of shape and weight. This is done at the same time as enhancing the other domains for self-evaluation and it involves tackling body checking, body avoidance, and feeling fat.

##### Addressing body checking and avoidance

Patients are often not aware that they are engaging in body checking and that it is maintaining their body dissatisfaction. The first step is therefore to obtain detailed information about their checking behavior by asking patients to monitor it. Patients are then educated about the adverse effects of repeated body checking as the way in which they check tends to provide biased information that leads them to feel dissatisfied. For example, scrutinizing parts of one's body magnifies apparent defects, and only comparing oneself to thin and attractive people leads one to draw the conclusion that one is unattractive. Most patients need substantial and detailed help to curb their repeated body checking and invariably attention needs to be devoted to their mirror use.

Patients who avoid seeing their bodies also need considerable help. They should be encouraged to progressively get used to the sight and feel of their body. This may take many successive sessions.

##### Addressing “feeling fat”

“Feeling fat” is an experience reported by many women but the intensity and frequency of this feeling appears to be far greater among people with eating disorders. Feeling fat is a target for treatment because it tends to be equated with being fat (irrespective of the patient's actual shape and weight) and hence maintains body dissatisfaction. Although this topic has received little research attention, clinical observation suggests that feeling fat is a result of mislabeling certain emotions and bodily experiences. Consequently, patients are helped to identify the triggers of their feeling fat experiences and the accompanying feelings. These typically are negative mood states (eg, feeling bored or depressed) or physical sensations that heighten body awareness (eg, feeling full, bloated, or sweaty). Patients are then helped to view “feeling fat” as a cue to ask themselves what else they are feeling at the time and once recognized to address it directly.

#### Exploring the origins of overevaluation

Toward the end of Stage three it is often helpful to explore the origins of the patient's sensitivity to shape, weight, and eating. A historical review can help to make sense of how the problem developed and evolved, highlight how it might have served a useful function in its early stages, and the fact that it may no longer do so. If a specific event appears to have played a critical role in the development of the eating problem, the patient should be helped to reappraise this from the vantage point of the present. This review helps patients distance themselves further from the eating disorder frame of mind or “mindset.”

### Addressing Dietary Rules

Patients are helped to recognize that their multiple extreme and rigid dietary rules impair their quality of life and are a central feature of the eating disorder. A major goal of treatment is therefore to reduce, if not eliminate altogether, dieting. The first step in doing so is to identify the patient's various dietary rules together with the beliefs that underlie them. The patient is then helped to break these rules to test the beliefs in question and to learn that the feared consequences that maintain the dietary rule (typically weight gain or binge eating) are not an inevitable result. With patients who binge eat, it is important to pay particular attention to “food avoidance” (the avoidance of specific foods) as this is a major contributory factor. These patients need to systematically re-introduce the avoided food into their diet.

### Addressing Event-related Changes in Eating

Among many patients with eating disorders, eating habits change in response to outside events and changes in their mood. The change may involve eating less, stopping eating altogether, overeating, or binge eating. If these changes are prominent, patients need help to deal directly with the triggers. Generally this may be achieved by training them in “proactive” problem solving coupled with the use of functional means of modulating mood.

### Addressing Clinical Perfectionism, Low Self-esteem, and Interpersonal Problems

As noted earlier, there are 2 main forms of CBT-E. The components of the focused version are described previously. The “broad” version also includes these strategies and procedures but, in addition, addresses one or more “external” (to the core eating disorder) processes that may be maintaining the eating disorder. It is designed for patients in whom clinical perfectionism, core low self-esteem, or marked interpersonal problems are pronounced and appear to be contributing to the eating disorder. If the therapist decides, in the review of progress (Stage two), to use one or more of these modules, they should become a major component of all subsequent sessions. In the original version of the broad form of CBT-E a fourth module, “mood intolerance,” was included but this has since been integrated in to the standard, focused, form of the treatment as part of addressing events and moods. A description of the main elements of the 3 modules follows. A more detailed account is available in the main treatment guide.^32^

#### Addressing clinical perfectionism

The psychopathology of clinical perfectionism is similar to that of an eating disorder.^33^ Its core is the overevaluation of striving to achieve and achievement itself. People with clinical perfectionism judge themselves largely, or exclusively, in terms of working hard toward, and meeting, personally demanding standards in areas of life that they value. If they have a coexisting eating disorder such extreme standards are applied to their eating, weight, and shape. This intensifies key aspects of the eating disorder including dietary restraint, exercise, and shape checking. It is usually evident from the patient's behavior and it can interfere with important aspects of treatment, leading to, for example, overly detailed recording and a strong resistance to relaxing dietary restraint.

The strategy for addressing clinical perfectionism mirrors that used to address the overevaluation of shape and weight and the two can be addressed more or less at the same time. The first step is to add perfectionism to the patient's formulation and to consider the consequences of this for the patient and his or her life, including the self-evaluation pie-chart. Patients are then encouraged to take steps to enhance the importance of other, nonperformance related, domains for self-evaluation.

It is helpful to consider collaboratively patients' goals in areas of life that they value, which are usually multiple, rigid, and extreme, and whether these goals are in fact counterproductive and impairing their actual performance. Performance checking is addressed similarly to shape checking, beginning by first asking patients to record times when they are checking their performance. Then the therapist helps them appreciate that the data they obtain is likely to be skewed as a result of using biased assessment processes, such as selective attention to failure. Avoidance and procrastination also need to be addressed, as they interfere with patients being able to assess their true ability with the result that their fears of failure are maintained.

#### Addressing core low self-esteem

People with core low self-esteem (CLSE) have a longstanding and pervasive negative view of themselves. It is largely independent of the person's actual performance in life (ie, it is unconditional) and is not secondary to the presence of the eating disorder. The presence of CLSE results in the individual striving especially hard to control eating, weight, and shape to retain some sense of self-worth. It is generally a barrier to engaging in treatment as patients do not feel they deserve treatment nor do they believe that they can benefit from it.

If it is to be directly addressed in treatment, it is added to the patient's formulation in Stage two and tackled alongside, although slightly later than, the steps addressing the overevaluation of shape and weight. This involves educating patients about the role of CLSE in maintaining the eating disorder and contributing to other difficulties in their life. Patients are helped to identify and modify the main cognitive maintaining processes, including discounting positive qualities and the overgeneralization of apparent failures. Previous views of the self are reappraised, using both cognitive restructuring and behavioral experiments, to help patients to reach a more balanced view of their self-worth.

#### Addressing interpersonal problems

Interpersonal problems are common among patients with eating disorders, although they generally improve as the eating disorder resolves. Such problems may include conflict with others and difficulties developing close relationships. If these problems, and the resulting effects on mood, directly influence the patient's eating, they may be addressed through the use of proactive problem solving and functional mood modulation and acceptance (as described earlier). However, in some cases interpersonal problems powerfully maintain the eating disorder through a variety of direct and indirect processes or they interfere with treatment itself. Under these circumstances, they need to become a focus of treatment in their own right.

The strategy used in CBT-E is to use a different psychological treatment to achieve interpersonal change, namely Interpersonal Psychotherapy (IPT). This is an evidence-based treatment that helps patients identify and address current interpersonal problems. In style and content IPT is very different from CBT-E. For this reason it is not “integrated” with CBT-E as such: rather, each session has a CBT-E component and an IPT one. More detailed information about IPT and its use with patients with eating disorders is available in a recent book chapter.^34^

## Stage four

Stage four, the final stage in treatment, is concerned with ending treatment well. The focus is on maintaining the progress that has already been made and reducing the risk of relapse. Typically there are 3 appointments about 2 weeks apart. During this stage, as part of their preparation for the ending of treatment, patients discontinue self-monitoring and begin weekly weighing at home.

To maximize the chances that progress is maintained, the therapist and patient jointly devise a personalized plan for the following few months until a posttreatment review appointment (usually about 20 weeks later). Typically this includes further work on body checking, food avoidance, and perhaps further practice at problem solving. In addition, patients are encouraged to continue their efforts to develop new interests and activities.

There are 2 elements to minimizing the risk of relapse. First, patients need to have realistic expectations regarding the future. Expecting never to experience any eating difficulties again makes patients vulnerable to relapse because it encourages a negative reaction to even minor setbacks. Instead, patients should view their eating problem as an Achilles heel. The goal is that patients identify setbacks as early as possible, view them as a “lapse” rather than a “relapse,” and actively address them using strategies that they learned during treatment.

## Underweight patients

The strategies and procedures described so far are also relevant to patients who are underweight (mostly cases of anorexia nervosa but some cases of eating disorder NOS). However, CBT-E has to be modified to address certain characteristics of these patients.

The first priority is to address motivation, as often these patients do not view undereating or being underweight as a problem. This may be done in several ways and relies on a good therapeutic alliance. The patient is provided with a personalized education about the psychological and physical effects of being underweight. This helps them to understand that some of the things that they find difficult (eg, being obsessive and indecisive, being unable to be spontaneous, being socially avoidant, lacking sexual appetite) are a direct consequence of being a low weight rather than being a reflection of their true personality. The patient is helped to think through the advantages and disadvantages of change, including a consideration of how things are likely to be in the future if they choose not to change and how this would fit with their aspirations. The therapist shows intense interest in the patient as a person, beyond the eating disorder, and helps them to reflect on the state of all aspects of their life, including their relationships, their physical and psychological well-being, their work, and their personal values. The patient is encouraged to experiment with making changes to learn more about the pros and cons of their current behavior. The goal is for patients themselves to decide to regain weight rather than this decision being imposed by the therapist. If this is successful, it greatly assists subsequent weight regain.

Second, the undereating and the consequent state of starvation must be addressed. It is important to help patients to realize that undereating, and being underweight maintain the eating disorder and this is illustrated in a personalized formulation. Once the patient has agreed to regain weight it is explained that weight regain should be gradual and steady and that they should aim to maintain an average energy surplus of 500 calories each day to regain an average of 0.5 kg (1.1 lb) per week. The therapist helps the patient to devise and implement a daily plan of eating (which may be supplemented by energy-rich drinks) that meets this target.

Treatment needs to be extended from the typical 20 weeks to about 40 weeks to allow sufficient time for patients to decide to change, to reach a healthy weight, and then practice maintaining it. It can be helpful to involve others in the weight-gain process to facilitate the patient's own efforts. This is especially so with young patients who are living at home with their parents.

## Final comments

Hopefully it will be clear from this brief account of CBT for eating disorders that major advances have been made and are continuing to be made. Perhaps most prominent among these is the adoption of a transdiagnostic approach to treatment whereby treatment is no longer for a specific eating disorder (eg, bulimia nervosa) but is directed at eating disorder psychopathology and the processes that maintains it. As a result, an empirically supported treatment approach has evolved that is suitable for all forms of eating disorder and one that is highly individualized.

Many challenges remain. First and foremost, treatment outcome needs to be further improved, especially in the case of patients who are substantially underweight. Second, understanding more about the way in which treatment works, and the active ingredients of treatment, could inform the design of a more potent version. Doubtless some elements could be discarded whereas others may need to be enhanced.^35^ We need treatments that are effective and efficient. Last, we need to facilitate the dissemination of evidence-based practice. Many patients receive suboptimal treatment. There are several possible reasons for this but prominent among them is the fact that few therapists have received the necessary training.

## Figures and Tables

(Fairburn CG. Eating disorders: the transdiagnostic view and the cognitive behavioral theory. In: Fairburn CG. Cognitive behavior therapy and eating disorders. New York: Guilford Press; 2008. p. 7–22).

(Fairburn CG, Cooper Z, Shafran R, et al. Enhanced cognitive behavior therapy for eating disorders: the core protocol. In: Fairburn CG. Cognitive behavior therapy and eating disorders. New York: Guilford Press; 2008. p. 47–193.)

(Fairburn CG, Cooper Z, Shafran R, et al. Enhanced cognitive behavior therapy for eating disorders: the core protocol. In: Fairburn CG. Cognitive behavior therapy and eating disorders. New York: Guilford Press; 2008. p. 47–193.)

## References

[1] National Institute for Clinical Excellence (NICE) (2004). Eating disorders—core interventions in the treatment and management of anorexia nervosa, bulimia nervosa and related eating disorders. http://www.nice.org.uk.

[2] Fairburn C.G., Cooper Z., Doll H.A. (2009). Transdiagnostic cognitive behavioral therapy for patients with eating disorders: a two-site trial with 60-week follow-up. Am J Psychiatry.

[3] American Psychiatric Association (1994). Diagnostic and statistical manual of mental disorders.

[4] Fairburn C.G., Bohn K. (2005). Eating disorder NOS (EDNOS): an example of the troublesome “not otherwise specified” (NOS) category in DSM-IV. Behav Res Ther.

[5] Dalle Grave R., Calugi S. (2007). Eating disorder not otherwise specified on an inpatient unit. Eur Eat Disord Rev.

[6] Miller G., Holden C. (2010). Proposed revisions to psychiatry's canon unveiled. Science.

[7] Fairburn C.G., Cooper Z., Bohn K. (2007). The severity and status of eating disorder NOS: implications for DSM-V. Behav Res Ther.

[8] Wilson G.T., Grilo C.M., Vitousek K.M. (2007). Psychological treatment of eating disorders. Am Psychol.

[9] Shapiro J.R., Berkamn N.D., Brownley K.A. (2007). Bulimia nervosa treatment: a systematic review of randomized controlled trials. Int J Eat Disord.

[10] Hay P.P.J., Bacaltchuk J., Stefano S. (2009). Psychological treatments for bulimia nervosa and binging. Cochrane Database Syst Rev.

[11] Fairburn C.G., Jones R., Peveler R.C. (1993). Psychotherapy and bulimia nervosa: the longer-term effects of interpersonal psychotherapy, behaviour therapy and cognitive behaviour therapy. Arch Gen Psychiatry.

[12] Agras W.S., Walsh B.T., Fairburn C.G. (2000). A multicenter comparison of cognitive behavioral therapy and interpersonal psychotherapy for bulimia nervosa. Arch Gen Psychiatry.

[13] Wilson G.T., Fairburn C.G., Nathan P.E., Gorman J.M. (2007). Treatments for eating disorders. A guide to treatments that work.

[14] Fairburn CG. Transdiagnostic CBT for eating disorders “CBT-E”, presented at association for behavioral and cognitive therapy. New York; 2009.

[15] Mitchell J., Devlin M., de Zwaan M. (2008). Binge eating disorder. Clinical foundations and treatment.

[16] Brownley K.A., Berkman N.D., Sedway J.A. (2007). Binge eating disorder treatment: a systematic review of randomized controlled trials. Int J Eat Disord.

[17] Sysko R., Walsh T. (2008). A critical evaluation of the efficacy of self-help interventions for the treatment of bulimia nervosa and binge-eating disorder. Int J Eat Disord.

[18] Wilson G.T., Wilfley D.E., Agras W.S. (2010). Psychological treatments of binge eating disorder. Arch Gen Psychiatry.

[19] Fairburn C.G., Cooper Z., Shafran R. (2003). Cognitive behaviour therapy for eating disorders: a “transdiagnostic” theory and treatment. Behav Res Ther.

[20] Fairburn C.G., Harrison P.J. (2003). Eating disorders. Lancet.

[21] Fairburn C.G., Cooper Z., Cooper P., Brownwell K.D., Foreyt J.P. (1986). The clinical features and maintenance of bulimia nervosa. Physiology, psychology and treatment of eating disorders.

[22] Cooper Z., Stewart A., Fairburn C.G. (2008). CBT-E and the younger patient. Cognitive behavior therapy and eating disorders.

[23] Dalle Grave R., Bohn K., Hawker D., Fairburn C.G. (2008). Inpatient, day patient, and two forms of outpatient CBT-E. Cognitive behavior therapy and eating disorders.

[24] Dalle Grave R, Fairburn CG. Intensive CBT for eating disorders. New York: Guilford Press, in press.

[25] Fairburn C.G. (2008). Cognitive behavior therapy and eating disorders.

[26] Fairburn C.G., Cooper Z., Waller D., Fairburn C.G. (2008). The patients: their assessment, preparation for treatment and medical management. Cognitive behavior therapy and eating disorders.

[27] Fairburn C.G., Agras W.S., Walsh B.T. (2004). Prediction of outcome in bulimia nervosa by early change in treatment. Am J Psychiatry.

[28] Agras W.S., Crow S.J., Halmi K.A. (2000). Outcome predictors for the cognitive-behavioral treatment of bulimia nervosa: data from a multisite study. Am J Psychiatry.

[29] Fairburn C.G. (1995). Overcoming binge eating.

[30] Schmidt U., Treasure J. (1993). Getting better bit(e) by bit(e). A survival guide for sufferers of bulimia nervosa and binge eating disorders.

[31] Fairburn C.G., Cooper Z., Shafran R., Fairburn C.G. (2008). Enhanced cognitive behavior therapy for eating disorders: the core protocol. Cognitive behavior therapy and eating disorders.

[32] Fairburn C.G., Cooper Z., Shafran R., Fairburn C.G. (2008). Clinical perfectionism, core low self-esteem and interpersonal problems. Cognitive behavior therapy and eating disorders.

[33] Shafran R., Cooper Z., Fairburn C.G. (2002). Clinical perfectionism: a cognitive-behavioural analysis. Behav Res Ther.

[34] Murphy R., Straebler S., Cooper Z., Dancyger I.F., Fornari V.M. (2009). Interpersonal psychotherapy (IPT) for eating disorders. Evidence based treatments for eating disorders.

[35] Kazdin A.E., Nock M.K. (2003). Delineating mechanisms of change in child and adolescent therapy: methodological issues and research recommendations. J Child Psychol Psychiatry.

